# DR and SPIT: Statistical approaches for identifying transient structure in intrinsically disordered proteins via NMR chemical shifts

**DOI:** 10.1002/pro.70250

**Published:** 2025-08-15

**Authors:** Dániel Kovács, Andrea Bodor

**Affiliations:** ^1^ Analytical and BioNMR Laboratory, Institute of Chemistry ELTE, Eötvös Loránd University Budapest Hungary; ^2^ Hevesy György PhD School of Chemistry, Institute of Chemistry ELTE, Eötvös Loránd University Budapest Hungary

**Keywords:** binomial test, intrinsically disordered proteins, NMR spectroscopy, secondary chemical shifts, secondary structural propensity, *t*‐statistics

## Abstract

Intrinsically disordered proteins (IDPs) play key roles in various biological processes; they are associated with liquid–liquid phase separation and are targets in disorder‐based drug design. Efforts to identify their structural propensities—that can be linked to molecular recognition, malfunction, targeting—still lead to ambiguous results. Secondary structure is routinely assessed by NMR spectroscopy by calculating the secondary chemical shifts (SCSs). Focusing on a given environment in the polypeptide backbone, SCSs highlight the deviation from the “random coil” state. However, the analysis is dependent on which of the numerous random coil chemical shift (RCCS) predictors is applied in the calculations, resulting in an especially pronounced ambiguity for IDPs. To overcome this, we introduce two novel statistical tools that enable the sound identification of structural propensities. We propose the chemical shift discordance ratio (DR) for prefiltering RCCS predictors based on self‐consistency. Further on, we introduce the Structural Propensity Identification by *t*‐statistics (SPIT) approach for extracting maximum information from SCS data by using multiple RCCS predictors simultaneously. This way SCS patterns indicating structural propensities can be clearly distinguished from the “noise”. The applicability of these methods is demonstrated for four proteins of varying degrees of disorder. Ubiquitin and α‐synuclein are used as respective benchmarks for a globular and a disordered protein, while two proline‐rich IDPs are included as especially challenging molecules in secondary structure analysis.

## 1 INTRODUCTION

IDPs constitute a large part of the proteome; they have diverse biological roles and are connected to various diseases (Chakrabarti & Chakravarty, 2022; Peng et al., 2015; Weber, 2016). IDPs lack stable 3‐dimensional structure; instead, they are present as rapidly interchanging conformational ensembles (Marsh & Forman‐Kay, 2009; Mukrasch et al., 2009; Ono et al., 2015). Consequently, it is difficult to highlight the regions with structural preferences that can be aimed at by drug candidate molecules. Despite obvious pharmaceutical implications, targeting IDPs remains a challenge in structure‐based drug design (Robustelli et al., 2022; Saurabh et al., 2023; Wyss & Zartler, 2020). To overcome this issue, here we propose an approach to confidently pinpoint the structural propensities in IDPs, using two statistical methods based on atomic level characterization by NMR measurements.

**FIGURE 1:**
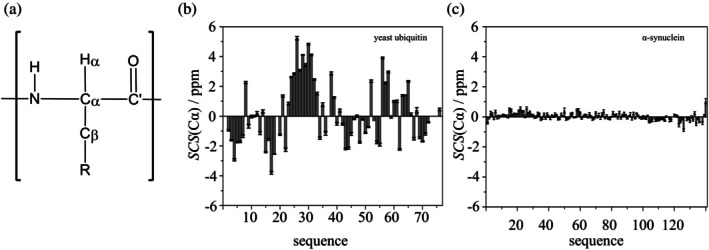
(a) General representation of an amino acid residue in the polypeptide backbone, highlighting the different atom types, while “R” represents all side chain atoms except Cβ. (b) The Cα SCSs of yeast ubiquitin calculated as the average of values using seven RCCS predictors. (c) The Cα SCSs of α‐synuclein calculated as the average of seven RCCS predictors. In (b and c) the whiskers represent the standard error.

As disorder defies both X‐ray diffraction (XRD) and cryo‐electron microscopy, currently, NMR spectroscopy is the only experimental technique capable of reporting on IDP structural propensities in solution. Other techniques like circular dichroism spectroscopy as well as small angle X‐ray or neutron scattering can be used for global characterization of a biomolecule, while atomic level information is provided by NMR. Protein backbone (N, Cα, C′, HN, Hα) and side‐chain Cβ environments (Figure 1a) are sensitive to the distribution of backbone dihedral angles, thus, to the local secondary structure of the polypeptide (Spera & Bax, 1991) Consequently, the experimentally determined chemical shift values (*δ*_m_) of these atom types can be used for secondary structure analysis. Measured values for a given atom type are converted to secondary chemical shifts (SCSs) considering also the random coil chemical shift values (RCCSs). Thus, for the *i*th position in the amino acid sequence:

(1) $${\mathrm{SCS}}_{i}=\delta_{m,i}-{\text{RCCS}}_{i}$$

Further on, protein secondary structure is revealed from SCS data trends along the amino acid sequence either by visual inspection or by further SCS‐derived quantities (Berjanskii & Wishart, 2008; Camilloni et al., 2012; Hafsa et al., 2015; Marsh et al., 2006; Nielsen & Mulder, 2020; Tamiola & Mulder, 2012; Wang & Jardetzky, 2002b; Wishart et al., 1992). As the random coil state is not unambiguously defined, various RCCS predictors were developed using different approaches, but none of these has become the golden standard. This means there is an unavoidable ambivalence in SCS values and in any related quantity; moreover, the selection of the RCCS predictor affects the identification of secondary structural propensities (Kovács & Bodor, 2023). For folded proteins this is not problematic, as SCS values are large (±6 ppm); but IDPs have small amplitudes (±1 ppm), comparable to the uncertainty of RCCS values. In such cases, RCCS prediction introduces a ‘noise’ in the calculation (Figure 1b,c), which might cover up or falsely create structural propensities.

Thus, SCS analysis of IDPs necessitates careful selection of RCCS predictors. To enable the sound assessment of structural propensities, we present a novel method based on statistical techniques and relying on multiple predictors—instead of a single one commonly applied. This integrative approach enables mitigating the individual limitations of different RCCS predictors while leveraging their individual merits. We introduce the chemical shift discordance ratio (DR)—an exploratory metric for RCCS predictors characterizing self‐consistency. Further on, we propose the structural propensity identification by *t*‐statistics (SPIT) using multiple RCCS predictors in unison to find structural propensities and motifs.

Our model proteins used as examples have been chosen to represent different types of proteins with chemical shift data available for various atom types. We selected K48R yeast ubiquitin (yUBI) to model typical globular proteins, while α‐synuclein (α‐syn) serves as a benchmark IDP. Furthermore, the disordered C‐terminal of the WASP interacting protein (WIPc) and a 60‐residue long segment of the N‐terminal of the tumor suppressor protein p53 (p53TAD^1–60^) have been chosen to demonstrate how the introduced methods perform on proline‐rich molecules, an especially challenging class of IDPs.

## 2 METHODS AND CALCULATION

### 2.1 Chemical shift data

The presented chemical shift data were downloaded from the Biological Magnetic Resonance Data Bank (BMRB) (Hoch et al., 2023). Relevant data regarding the model proteins are summarized in Table 1.

**TABLE 1:** Experimental conditions and characteristics of the studied proteins.

Full name	Short name	BMRB entry	Number of residues	pH	*T*/K
Yeast ubiquitin K48R (folded)	yUBI	4769	76	7.50	303
α‐synuclein (IDP)	α‐syn	18,857	140	7.10	283
Proline rich C‐terminal domain of the WASP interacting protein (IDP)	WIPc	18,265	114	5.10	298
Proline rich N‐terminal domain of the tumor suppressor p53 protein (IDP)	p53TAD^1–60^	52,877	60	5.80	313

### 2.2 RCCS and SCS calculation

Eight RCCS predictors were considered for the analysis, as described earlier in Kovács & Bodor, 2023 (De Simone et al., 2009; Kjaergaard et al., 2011; Kjaergaard & Poulsen, 2011; Nielsen & Mulder, 2018; Sanz‐Hernandez & de Simone, 2017; Tamiola et al., 2010; Wang & Jardetzky, 2002a; Wishart et al., 1995). The Schwarzinger predictor was excluded from SPIT calculations as it is valid for acidic pH conditions. The conceptual evolution and details of RCCS libraries are summarized in Table S1 (Kovács & Bodor, 2023). For brevity, we limit our discussion to the reliable (Harmat et al., 2021; Iwadate et al., 1999; Spera & Bax, 1991) Cα and Hα environments, but both our methods are easily generalized to other atom types, as discussed later. RCCS and SCS calculations were performed by in‐house R scripts and MS Excel.

### 2.3 Discordance ratio (DR) analysis

The effect of secondary structural motifs on the chemical shifts of the six canonical atom‐types is well‐established in the literature (Borcherds & Daughdrill, 2018) and is summarized in Table S2. In helices, Cα chemical shifts experience a deshielding effect and a shielding effect in strands, and for Hα atoms the tendency is the opposite. This means that for a single amino acid, the secondary structural propensities conveyed by SCSs of Cα and Hα environments are consistent only if the corresponding signs differ (discordance); in the case of the same sign (concordance) the information is inconsistent (Table 2).

**TABLE 2:** Potential cases of discordance and concordance for the signs of SCSs.

	SCS (Cα) <0	SCS (Cα) >0
SCS (Hα) < 0	− − negative concordance	− + discordance, helical
SCS (Hα) > 0	+ − discordance, extended	+ + positive concordance

*Note*: Green text indicates sensible (well‐intrepretable) cases of discordance. The red color indicates the mutual information content of the two atom‐types is confusing in such cases.

Thus, a perfect predictor is characterized by complete discordance and each concordance represents an error. The discordance ratio of Cα‐Hα pairs can be defined as:

(2) $${\mathrm{DR}}_{\mathrm{C\alpha}-\mathrm{H\alpha}}=\frac{n_{\text{discordant}}}{n_{\mathrm{C\alpha}-\mathrm{H\alpha}}}$$

where *n*_discordant_ and *n*_Cα‐Hα_ are the number of discordant and all available Cα‐Hα SCS pairs, respectively. An RCCS predictor with *DR* ~ 1.0 is considered good; values ~0.5 indicate that the predictor does not perform better in yielding consistent structural information than tossing a coin; and values ≪0.5 mean that the predictor generally produces contradictory information.

Thus, *DR*_Cα‐Hα_ characterizes the self‐consistency of RCCS predictors for a given protein, under given experimental conditions and for the two atom types concerned. Whether *DR* is significantly different from 0.5 can be decided by a binomial test as follows.

For a given protein with *n*_Cα‐Hα_ residues for which both Cα and Hα are assigned, an RCCS predictor produces *n discordant* discordances as a realization of the random variable *N discordant*.

Under the *H*_0_ null‐hypothesis, *N*_discordant_ is a binomially distributed random variable with *n*_Cα‐Hα_ number of trials and success probability parameter 0.5, that is *N*_discordant_ ~ *B*(*n*_Cα‐Hα_, 0.5). This is actually the basic scenario of a series of coin tosses where *DR*_Cα‐Hα_ is the experimental estimate of the probability parameter. Performing a two‐tailed test at significance level *α* (commonly α = 5%), *H*_0_ is accepted if α/2 < *F*(*DR*_Cα‐Hα_) < 1 − α/2, where *F*(*DR*_Cα‐Hα_) is the cumulative distribution function of the corresponding binomial distribution evaluated at the experimental value of *DR*_Cα‐Hα_. If this condition is not met, then *H*_0_ is rejected (Figure S1).

### 2.4 Identification of structural propensities by *t*‐statistics (SPIT) approach

While the presented discordance ratio allows the selection of RCCS predictors, separation of the structural propensities from “SCS noise” can be done using the SPIT approach. Based on SCSs by *k*_pred_ predictors at position *i* in the amino acid sequence, a *t*‐value can be calculated as:

(3) $$t_{i}=\frac{m_{i}\left(\mathrm{SCS}\right)}{s_{i}/\sqrt{k_{\text{pred}}}}$$

where the real—but unknown—SCS value is approximated by the *m*_*i*_(*SCS*) average of the *k*_pred_ SCS values; *s*_*i*_ is the corresponding standard deviation. The *t*_*i*_ ratio is a Student's *t*‐distributed variable with *ν* = *k*_pred_ − 1 degrees of freedom. The probability that the *i*th real SCS is non‐negative can be calculated as:

(4) $$P_{i}\left(+\right)=P\left({\mathrm{SCS}}_{\text{real},i}\geq 0\right)=F_{t,\nu }\left(t_{i}\right)=1-P_{i}\left(-\right)$$

where *F*_*t*,*ν*_(*t*_*i*_) is the cumulative distribution function of the Student's *t*‐distribution evaluated at the experimentally acquired *t*_*i*_ value. In simple terms, *P*_*i*_(+) is the probability with which the *i*th residue has positive SCS value, and thus bears helical propensity for the Cα environment and β‐propensity for the Cβ or Hα environments, etc. So, if, for example, for a Cα atom *P*_*i*_(+) = 0.96, we are 96% confident that the *i*th residue can/may contribute to a helical residual structure. *P*_*i*_(−) is the complementary probability of *P*_*i*_(+). Assuming the independence of neighboring SCS values, it is possible to calculate the probability of *n*_seq_ consecutive residues all simultaneously bearing positive SCS values as the product of the *n*_seq_ individual *P*_*i*_(+) values starting at the *i*_start_ position. This quantity is the positive student‐based probability product (*SBPP*(+), Equation 5) and it shows whether the available SCS data underpin the presence of a helical propensity in the studied region. Similarly, *SBPP*(−) for extended propensity is calculated according to Equation 6.

(5) $$\text{SBPP}\left(+\right)=\prod_{i=i_{\text{start}}}^{i=i_{\text{start}}+n_{\mathrm{seq}}}P_{i}\left(+\right)$$

(6) $$\text{SBPP}\left(-\right)=\prod_{i=i_{\text{start}}}^{i=i_{\text{start}}+n_{\mathrm{seq}}}P_{i}\left(-\right)$$

**FIGURE 2:**
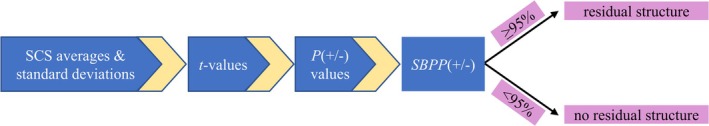
The schematic overview of the SPIT approach.

In accordance with the conventional significance level of 5%, we propose SBPP = 95% as the limit for identifying a structural propensity. Accordingly, only residues with *P*_*i*_(+) or *P*_*i*_(−) >95% contribute to a structural motif. The schematic overview of SPIT is shown in Figure 2, and a detailed calculation example can be found in Tables S3–S8.

Note that the independence of SCS obviously does not hold in case of a real structural propensity. SPIT is designed to detect deviation from complete disorder, where independence holds. This assumption is in fact an advantage, not a compromise, making SPIT the technique well suited to detect the slightest of SCS patterns that convey residual structure.

Based on assigning probability values to secondary structural propensities, SPIT is a new method, besides the existing secondary structural propensity (SSP) score (Marsh et al., 2006) and the δ2D method (Camilloni et al., 2012). However, both SSP and δ2D are conceptually different from SPIT because these techniques use a single RCCS dataset: that of RefDB (Zhang et al., 2002) and Camcoil (De Simone et al., 2009), respectively. It is possible to calculate SSP and δ2D with different RCCS libraries, but this yields various SSP and δ2D plots, which ultimately have to be compared and assessed together. SPIT enables using multiple RCCS libraries simultaneously, and it also inherently includes and deals with the uncertainty of RCCS values. Also, SPIT enables evaluating whether a certain propensity is present in an exact set of amino acids and does not only assign individual probability values to single residues. Furthermore, SPIT is by design more suited for distinguishing SCS patterns from noise than its counterparts. On the other hand, in contrast with SSP and δ2D SPIT does not evaluate the strength of structural propensities. The SSP and δ2D results of the four model proteins are compared and discussed in terms of SPIT in Figure S2.

In the following, we present the practical application of the presented approaches by showing how DR analysis can be used for RCCS dataset comparison and SPIT for structural propensity identification on the four studied model proteins.

## 3 RESULTS AND DISCUSSION

### 3.1 Discordance ratio (DR) analysis

**FIGURE 3:**
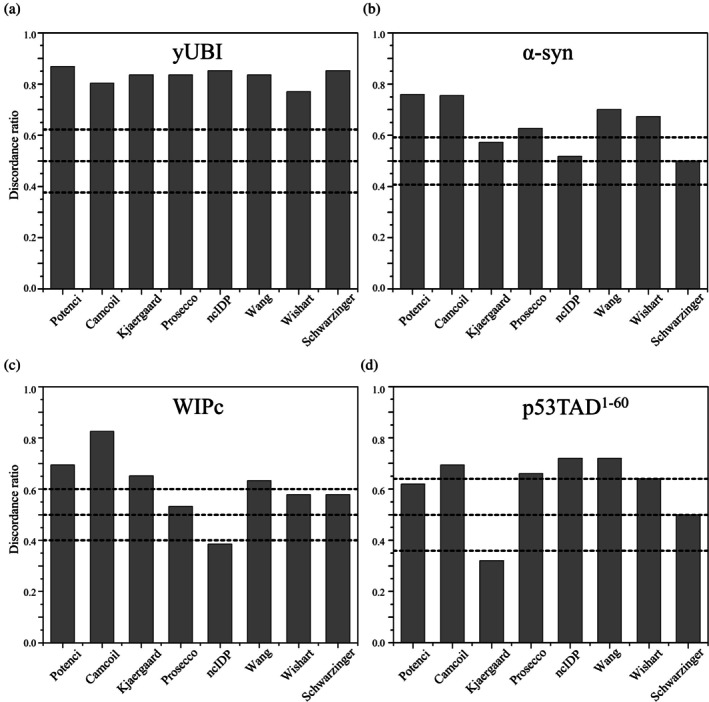
Discordance ratio (*DR*_Cα‐Hα_) values of the eight selected RCCS predictors for the model proteins. a) yUBI, b) α‐syn, c) WIPc, d) p53TAD^1–60^. The black horizontal lines from top to bottom represent the 97.5%, 50% and 2.5% probability limits of the corresponding binomial distribution. The *DR*_Cα‐Hα_ value of predictors between the upper and lower lines are not significantly different from 0.5, corresponding to mere chance. *DR*_Cα‐Hα_ values below the lower limit and above the upper limit are significantly lower and higher than 0.5, respectively.

We tested the selected eight RCCS predictors with respect to structural self‐consistency for a set of model proteins. As seen in Figure 3a, for the globular yUBI, all *DR*_Cα‐Hα_ values are similarly high, around 0.80. Differences between them are small—as for the secondary structural elements, the signs of both Cα and Hα SCSs are unambiguous. *DR*_Cα‐Hα_ values remain below 0.9, due to the presence of short loops, characterized by small, noise‐like SCSs. In addition, residues delimiting secondary structural motifs have irregular SCS patterns leading to concordant Cα‐Hα pairs. On the other hand, analyzing the data for the IDP α‐synuclein (Figure 3b) *DR*_Cα‐Hα_ values are generally smaller than for the globular protein; also, inter‐predictor differences are larger. *DR*_Cα‐Hα_ values of the Schwarzinger, Kjaergaard, and ncIDP predictors fall in the insignificant region, meaning that their Cα and Hα SCS patterns are in poor agreement with one another. As a conclusion, under these experimental conditions, for α‐synuclein, the remaining five predictors should be preferred in the SCS analysis.

In the case of the proline‐rich WIPc (Figure 3c) the calculated *DR*_Cα‐Hα_ values report on all three types of predictors: those significantly worse (ncIDP), significantly better (Camcoil, Potenci, Kjaergaard) and those not significantly different from a series of coin tosses (Prosecco, Wishart, Schwarzinger). Camcoil seems to convincingly outperform all other alternatives with respect to producing Cα‐Hα discordance. Selection based on *DR*_Cα‐Hα_ would result in keeping four predictors in the case of WIPc.

Regarding p53TAD^1–60^ (Figure 3d), the average *DR*_Cα‐Hα_ value is similar to those of the other two presented IDPs, although the pattern of individual values is unique. Interestingly, ncIDP and the wang library achieve the best performance with this protein, while RCCSs by the Kjaergaard method are significantly worse than randomness. This highlights the importance of always using multiple RCCS datasets and possibly atom types for the sound identification of transient structure in IDPs. Note that the small number of assigned chemical shift pairs (50 out of 60) raises the discordance ratio limit for significance to about 0.64, in contrast with 0.59 in the case of α‐syn (with 110 out of 140) and WIPc (with 109 out of 114) assigned chemical shift pairs. This is a completely natural mathematical phenomenon because the statistical power of the binomial test increases with the number of assigned chemical shifts and thus obviously with the size of the protein.

### 3.2 The SPIT approach for structural propensity identification

#### 3.2.1 Yeast ubiquitin K48R (yUBI)

**FIGURE 4:**
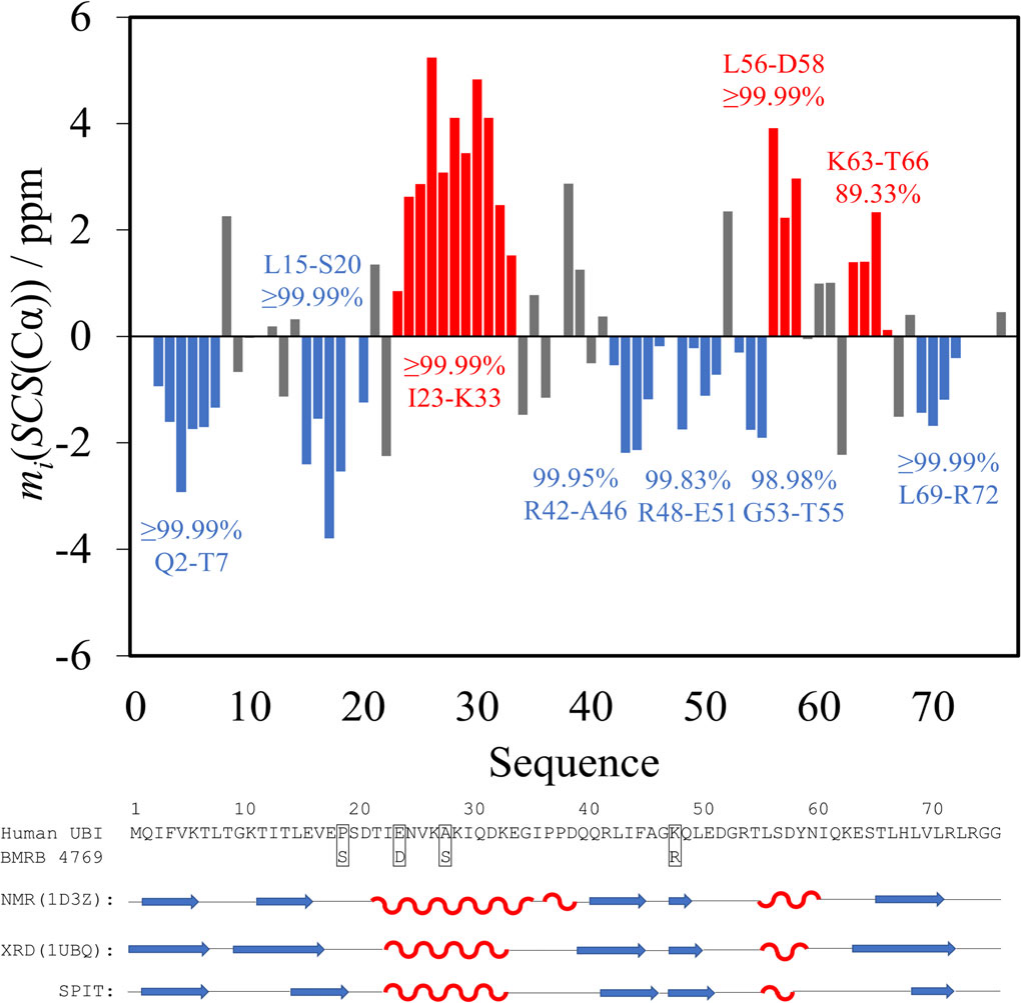
Average Cα SCS plot of yUBI. Regions of interest in SBPP calculation are highlighted in red for helical and in blue for extended propensities. The sequence and secondary structure of human ubiquitin (according to 2 PDB datasets) and yUBI (according to SPIT) are overlaid.

The SPIT results show that almost all SBPP(−) and SBPP(+) values are >95%, a clear indication of structural propensities (Figure 4), and in accordance with the motifs from PDB structures of human ubiquitin determined by different methods (1D3Z: NMR, 1UBQ: XRD). As pointed out earlier, the PDB structures have slight differences regarding the location and length of secondary structural motifs (Kovács & Bodor, 2023). Nevertheless, the first β‐sheet starting at Q2, which terminates at T7 according to 1UBQ and at K6 in the 1D3Z structure, is clearly identified by SPIT; the Q2‐T7 segment has an SBPP(−) >99.99%. Differences between the SPIT results and the two PDB structures are observed in the L8‐S20 region, as the secondary structures of yeast and human ubiquitin differ because of the P19S mutation.

For 1D3Z and 1UBQ, β‐sheets are identified between T12–E16 and G10–V17, respectively, while SPIT indicates the sheet very convincingly at L15–S20. Besides, the K23–K33 helix identified by SPIT exactly matches the 1UBQ structure, while in the 1D3Z structure the motif starts at T22. The missing assignment for P37 makes the identification of the short P37–D39 helical motif in 1D3Z impossible by SPIT. The Q40–E51 region harbors two shorter, separate β‐motifs. SPIT identifies these at R42–A46 and R48–E51 with SBPPs >99%. The first motif starts at Q40 for 1UBQ and at Q41 for 1D3Z, both last until F45; while the second one spans K48–L50 (1UBQ) or K48–Q49 (1D3Z). Note that a 2‐residue sheet is physically not viable. SPIT highlights an L56–D58 helical motif, which also spans Y59 and N60 according to 1UBQ and 1D3Z, respectively. The two PDB structures show considerable differences regarding the C‐terminal sheet: in 1UBQ, the E64–R72 region is involved, while for 1D3Z only the T66–L71 region, and SPIT indicates only the L69–R72 segment. Meanwhile, SCSs of the K63–T66 region are positive, thus excluding a sheet; but the SBPP(+) is only 89.33%, therefore, no helix is identified either. Altogether, SPIT locates the known secondary structural motifs of yUBI with great certainty. Differences from the two PDB structures are often smaller than those between the two structures themselves or are explained by the slight sequential differences between yUBI and human ubiquitin.

#### 3.2.2 α‐Synuclein (α‐syn)

**FIGURE 5:**
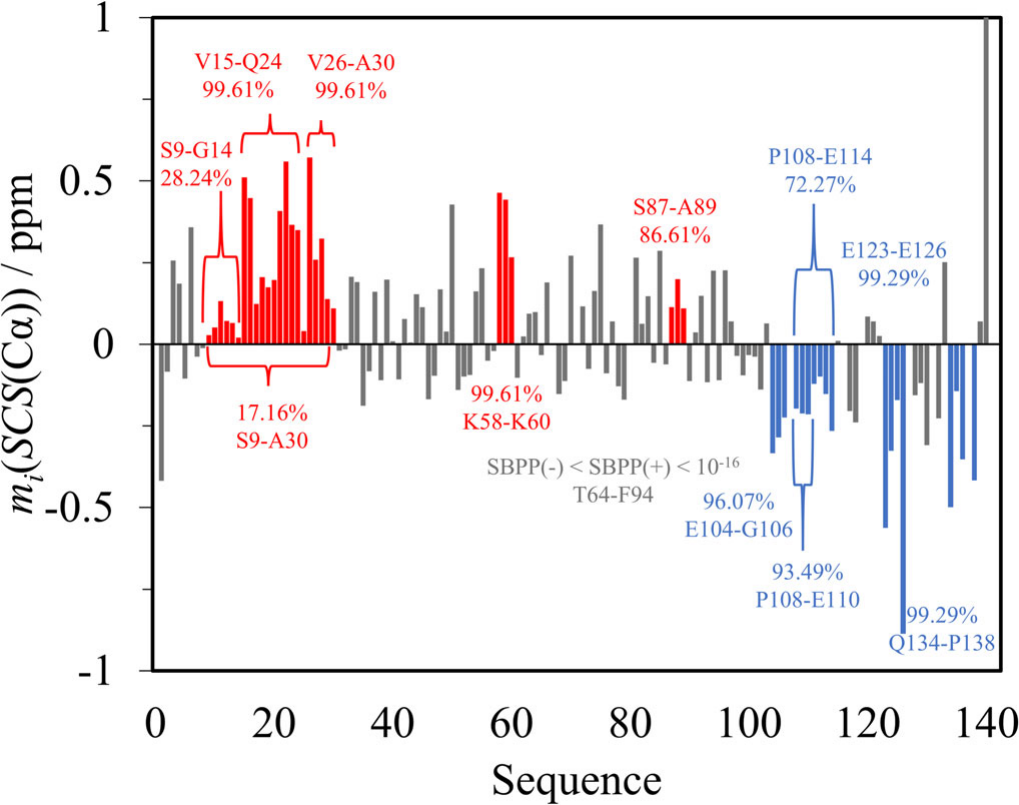
Average Cα SCS plot of α‐synuclein. Regions of interest are highlighted in red for helical and in blue for extended propensities. Results related to the T64‐F94 region—especially chosen for demonstrative purposes regarding complete disorder—are shown in gray. Where multiple shorter stretches of a region are analyzed, curly brackets support visualization.

Figure 5 shows the application of SPIT to α‐synuclein. Even though in the S9‐A30 region the average SCSs are all positive, the calculated SBPP(+) is only 17.16%—indicating that the existence of a nascent helix is unlikely. Limiting the calculation to V15‐Q24 and V26‐A29, the result is 97.18% and 99.61%, suggesting two helical propensity segments, separated by the G25 residue, belonging to neither region.

Further on, the K58‐K60 segment has an SBPP(+) of 99.61%, qualifying as a helical propensity. While the shortest 3–10 helices are known to be only three residues long (Pal et al., 2003), assuming the presence of such a small nascent helix in an IDP requires further evidence, highlighting that SPIT—as any statistical method—must be interpreted in an experimental and physical context not merely in itself. Also, the risk of false positive identification increases as the number of included residues decreases. The T64‐F94 region with respective SBPP(+) and SBPP(−) values of 1.475 × 10^−17^ and 1.835 × 10^−26^ shows how SPIT correctly indicates the lack of order when applied to SCSs with non‐uniform sign. The E104‐G106 segment at the C‐terminus has an SBPP(−) of 96.07%, but as the assignment for A107 is missing, no reliable conclusion can be drawn. For the P108‐E114 and P108‐E110 segments, the respective SBPP(−) values are only 72.27% and 93.49%, indicating no residual extended structure. On the other hand, in the E123–E126 stretch, the 99.29% SBPP(−) indicates a β‐propensity. The missing assignment for M127 makes the determination of the propensity length difficult. P128 does not contribute as its *P*_*i*_(−) value is only 90.82%. Finally, although E137 lacks assignment, for the Q134‐P138 segment, an SBPP(−) of 96.39% indicates a nascent extended structure. Note that although a missing assignment evidently weakens the conclusions, it does not hinder SBPP calculation mathematically because the missing residue can simply be omitted during the final multiplication step. Overall, the SPIT results strengthen the earlier observations relating to the mild helical propensities in the N‐terminal region and the extended conformations of the C‐terminal region (Alderson & Markley, 2013; McClendon et al., 2009).

#### 3.2.3 Proline rich C‐terminal domain of the WASP interacting protein (WIPc)

**FIGURE 6:**
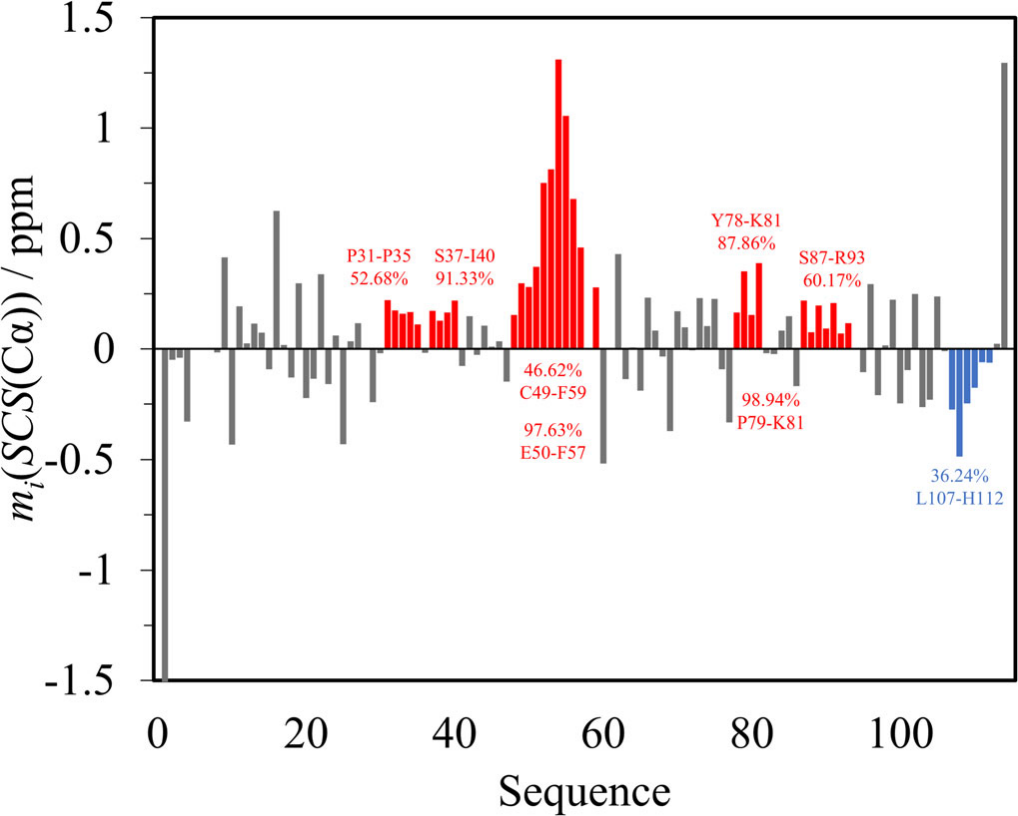
Average Cα SCS plot of WIPc. Regions of interest for SBPP calculation are highlighted in red for helical and in blue for extended propensities.

In their article describing the WIPc construct, Haba et al. (2013) state that the C49‐F59 segment has a notable helical propensity. This is based on SCSs calculated using ncIDP and three different online disorder predictors which rely only on the amino acid sequence and no experimental data for determining secondary structural propensities. The authors also note that disorder prediction especially emphasizes the helicity of the F57‐H60 segment. SPIT analysis with the seven selected RCCS datasets refines this result (Figure 6). The average SCS of H60 is negative, so this residue is excluded from the helical motif. The SBPP(+) value for the entire C49‐F59 region is only 46.62%, so the nascent helicity of the entire segment is improbable. The *P*_*i*_(+) for Y58 is 50.62%, which breaks the trend despite the corresponding *P*_*i*_(+) value of 99.90% for F59. So, based on SPIT, the helical motif may only be extended up to F57. For P48‐F57, the SBPP(+) value is only 76.17%; also, the value calculated for the C49‐F57 region—suggested helical by Haba et al.—is 92.13%, still below 95%. Finally, checking the E50‐F57 stretch, an SBPP(+) of 97.63% is obtained. Thus, based on SPIT analysis, we suggest the presence of a helical propensity in the E50‐F57 region. Nevertheless, depending on the further use and context of the analysis, the longer C49‐F59 could still be hypothesized to harbor helical propensity, for example, in a docking study. Especially as the risk of false negative identification increases with the number of included residues. However, SPIT calls attention to critical amino acids and raises the critical awareness of the investigator even in such cases.

Haba et al. also proposed a transient N‐capped helix between K76 and S80. As the average SCSs of K76 and S77 are negative, this is not implied by the data. However, the Y78‐K81 stretch exhibits an SBPP(+) of 87.86%, while the corresponding value for the P79‐K81 region is 98.94%. So, if anywhere, the N‐capped helix should be placed between Y78 and K81, although the SBPP value below 95% is not very convincing.

A nascent helicity has also been proposed for the S87‐E95 segment. In this region, the longest stretch of consecutive positive average SCSs is between S87 and R93 with an SBPP(+) of only 60.17%. Even for the S87‐S89 region, we only get 90.07%.

The P31–P36 region is special because it contains only proline residues. Therein, the P31–P35 stretch has consecutive positive average SCSs but an SBPP(+) of only 52.68%. This region might have a transient poly‐proline helical propensity, but SPIT highlights that different RCCS predictors are not very consensual on this motif. Therefore, the statistical analysis suggests this motif is highly unlikely to have the suggested helical tendency, taking RCCS uncertainty and measured chemical shift information into account.

Other proline‐rich stretches of the sequence are P17–P23, P66–P70, and P99–P105. Haba et al. note that these are characterized by SCSs of alternating signs in Cα based on ncIDP. These alternating patterns are also present in Figure 6. Thus, identification of a poly‐proline motif is more soundly based on multiple RCCS predictors. However, there is no point in calculating SBPPs because a pattern lacking consecutive SCSs of the same sign is strictly outside the scope of SPIT.

The consecutive negative average SCSs for L107‐H112 have no practical significance, as they all belong to a terminal expression tag. Also, no corresponding SBPP(−) value exceeding the 95% limit can be found in this region.

In conclusion, SPIT suggests that the helical motif in the middle region of the construct is shorter than suggested by Haba et al.; nevertheless, the latter part of this helical segment is part of the Wiskott‐Aldrich syndrome protein (WASP) binding site, and it contributes to the intermolecular interaction.

#### 3.2.4 Proline rich N‐terminal domain of the tumor suppressor p53 protein (p53TAD^1^ ^–60^)

**FIGURE 7:**
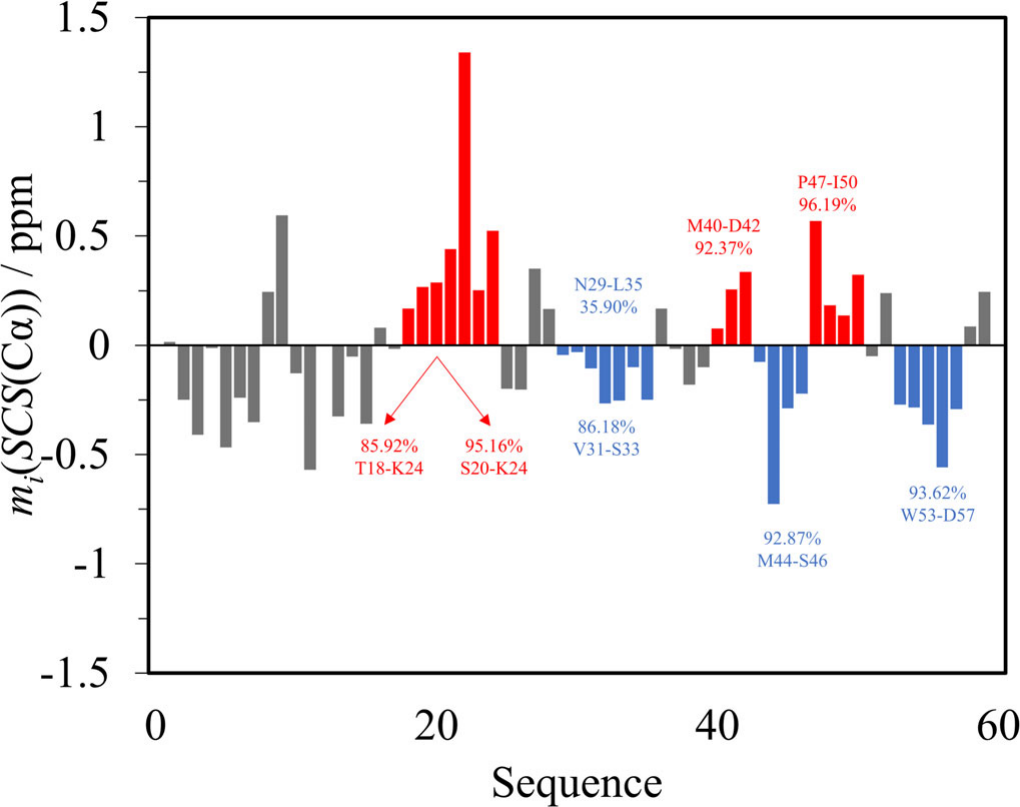
Average Cα SCS plot of p53TAD^1–60^. Regions of interest are in red for helical and in blue for extended propensities.

In the case of p53TAD^1–60^, we discuss the behavior of the regions implicated in harboring structural propensities by an earlier publication (Dudas et al., 2020) and those possessing longer sets of same‐sign SCSs (Figure 7).

In p53TAD^1–60^, residues T18‐K24 yield an SBPP(+) of 85.92%, a value not very convincing in terms of a transient helical structure. This is because the *P*_*i*_(+) values for T18 and W23 are 90.71 and 95.26%, respectively. For the F20‐K24 stretch, SBPP(+) increases to 95.16%, indicating transient helicity.

In the article of Dudas et al., the D41‐L43 segment was assumed to possess some weak helical propensity. The SPIT analysis yields an SBPP(+) value of 92.37% for the M40‐D42 stretch, which does not reach the 95% limit. However, some structural propensity is likely to be present between P47 and I50, which was originally suggested at residues D49‐E51. The corresponding SBPP(+) value is 96.19%.

The P13‐Q16 and W53‐D57 stretches could harbor some extended structural motif; however—in accordance with general disorder—none of these or any other segment in the protein produces SBPP values larger than 95%, thus no very convincing propensities are present. The case of the N29‐L35 region is an example of rejecting visually appealing noise based on SPIT. Here, the SBPP(−) value of 35.90% is way below the recommended level of acceptance, although all average SCSs are negative.

### 3.3 Comments on the availability of chemical shifts for different atom‐types

Both presented techniques can be easily extended to various atom types. Discordance ratios might be calculated for the Cα‐Cβ, Cα‐C', or C′‐Hα pairs. For atom types that are expected to experience same‐sign chemical shift changes in secondary structural motifs—e.g., Cα‐C', or Hα‐Cβ pairs—not discordance but concordance ratios may be calculated, and the same binomial test can be applied.

The SPIT approach can be applied to any atom‐type exactly in the same manner as demonstrated for Cα, the interpretation of SBPP values being the only difference. For example, Cβ SBPP(−) indicates a helical and SBPP(+) indicates extended propensity. SPIT can also be applied to the difference of Cα and Cβ SCSs, a popular quantity in SCS analysis (Figure S3).

This versatility of the presented techniques is especially important as commonly applied protein NMR measurements can be time‐consuming, especially in the case of IDPs, which require increased resolution because of inherently small signal dispersion. Although 2D ^1^H–^15^N HSQC spectra are generally the basis of protein NMR studies, simple HN and N chemical shifts are heavily affected by measurement conditions and are inconvenient in SCS analysis. Therefore, Cα, Cβ, C′, and Hα chemical shifts are commonly preferred, data which result from the assignment of multidimensional spectra. The common experiments are amide ^1^H‐detected spectra, but alternative detection approaches like ^13^C‐detected (Felli & Pierattelli, 2022) or ^1^Hα‐detected (Bodor et al., 2020; Mäntylahti et al., 2010; Sebak et al., 2022) are also used. However, despite the advanced pulse sequences and the large amount of deposited data in the BMRB, access to complete backbone assignment for a polypeptide is still not common. Generally, HN, N, and Cα shifts are deposited, but the availability of Hα, C′, and Cβ shifts varies. Fortunately, the generality of the DR and SPIT mitigates the effect of such limitations.

## 4 CONCLUSIONS

We already called attention to two previously unrecognized key facts regarding the identification of residual secondary structural elements in IDPs (Kovács & Bodor, 2023). Firstly, that it is heavily influenced by the ambiguity of the RCCS dataset used and secondly, that because of the “noise‐like” character of IDP secondary chemical shift patterns, the problem itself is essentially statistical in nature. This study is part of the inevitable paradigm shift from simple visual inspection of SCS data towards statistical approaches. We demonstrated that the herein introduced DR and SPIT computational tools facilitate selecting the appropriate RCCS library and extracting structural information from IDP SCS data. The *DR* values proved useful as an exploratory technique comparing RCCS predictors based on self‐consistency and are also a useful tool against chemical shift mis‐referencing (Figure S4). The SPIT approach and its SBPP metric enable identifying protein regions possessing structural propensities with great clarity by differentiating these from “SCS noise” as approximated by the uncertainty related to RCCS values. This makes SPIT especially suited for studying IDP in contrast to alternative methods like SSP and δ2D which try to locate transient structure on the disorder–structure continuum, while SPIT analyzes whether NMR data support any residual structure at all. We have also shown that in the case of convincing structural propensities SPIT is in good agreement with its alternatives while it is more locally sensitive in some cases and less prone to certain artifacts as a result of involving multiple RCCS predictors. We included examples highlighting how SPIT should be interpreted in the context of physical and chemical considerations. The results prove that DR and SPIT can be applied throughout the order–disorder spectrum, including proline‐rich IDPs where RCCS library selection and locally precise assessment of SCSs are extremely important. We also explained the generalizability of the presented techniques to other atom types (Figures S3 and S4) to encourage the broadest scientific audience in IDP research to apply these calculations for their systems uncompromised by the NMR data available. The presented methods open a new pathway to more rigorous and sound identification of IDP secondary structural propensities based on NMR spectroscopy, and all materials necessary to perform the calculations are available for download. Therefore, DR and SPIT are useful additions to the field of IDP research and are expected to drive future RCCS predictor development while enhancing both the general description and the pharmaceutical targeting of IDPs.

## AUTHOR CONTRIBUTIONS

**Dániel Kovács:** Methodology; conceptualization; investigation; writing – original draft; validation; visualization; software; data curation. **Andrea Bodor:** Conceptualization; methodology; supervision; resources; project administration; writing – review and editing; funding acquisition.

## CONFLICT OF INTEREST STATEMENT

The authors declare no conflict of interest.

## Supporting information


**DATA S1.** Supporting information.
The amino acid sequence of the four model proteins, a detailed SPIT calculation example, further notes on the versatile applicability of the DR and SPIT approaches and summary tables of RCCS prediction techniques and chemical shift trends in helical and extended structures are available in a text document. It also contains the results of benchmarking SPIT against similar computational tools: SSP and δ2D. Document name:
DR_SPIT_SI_Rev.docx.
In addition, five Excel spreadsheets including the calculations for the presented four proteins and one ready‐made for users are supplied as supplementary information in the following files:
DR_SPIT_yUBI.xlsx; DR_SPIT_a_syn.xlsx; DR_SPIT_WIPc.xlsx; DR_SPIT_p53_TAD.xlsx; DR_SPIT_default.xlsx.
Excel spreadsheets including the calculations are supplied. A detailed SPIT calculation example and further notes on the versatile applicability of the DR and SPIT approaches are also available. R‐scripts for RCCS prediction and SCS calculation can be downloaded (abnmr.elte.hu).

## Data Availability

The data that support the findings of this study are available in the supporting material. Additionally, R‐scripts for RCCS prediction and SCS calculation are available either from the corresponding author upon reasonable request, or they can be downloaded from the https://abnmr.elte.hu/ homepage.
