# Supplementary material for: DR and SPIT: Statistical approaches for identifying transient structure in intrinsically disordered proteins via NMR chemical shifts
Source: Protein Sci. 2025 Aug 15;34(9):e70250. doi: 10.1002/pro.70250 (PMC12356139; doi:10.1002/pro.70250)
Supplement: Supplementary file 1 — DATA S1. Supporting information. The amino acid sequence of the four model proteins, a detailed SPIT calculation example, further notes on the versatile applicability of the DR and SPIT approaches and summary tables of RCCS prediction techniques and chemical shift trends in helical and extended structures are available in a text document. It also contains the results of benchmarking SPIT against similar computational tools: SSP and δ2D. Document name: DR_SPIT_SI_Rev.docx. In addition, five Excel spreadsheets including the calculations for the presented four proteins and one ready‐made for users are supplied as supplementary information in the following files: DR_SPIT_yUBI.xlsx; DR_SPIT_a_syn.xlsx; DR_SPIT_WIPc.xlsx; DR_SPIT_p53_TAD.xlsx; DR_SPIT_default.xlsx. Excel spreadsheets including the calculations are supplied. A detailed SPIT calculation example and further notes on the versatile applicability of the DR and SPIT approaches are also available. R‐scripts for RCCS prediction and SCS calculation can be downloaded (abnmr.elte.hu). [file PRO-34-e70250-s001.zip › DR_SPIT_SI_Rev.docx]

**DR and SPIT: statistical approaches for identifying transient structure in intrinsically disordered proteins via NMR chemical shifts**

Electronic supplementary material

**Dániel Kovács^1,2^, Andrea Bodor^1^**

*^1^ ELTE, Eötvös Loránd University, Institute of Chemistry, Analytical and BioNMR Laboratory*

*^2^ Eötvös Loránd University, Hevesy György PhD School of Chemistry*

*Pázmány Péter sétány 1/A, Budapest 1117, Hungary*

**Table of contents**

S1. Amino acid sequences of the model proteins used in discordance ratio (DR) and Structural Propensity Identification by t-statistics (SPIT) calculations

S2. Existing RCCS predictors in the scientific literature

S3. Tabular summary of chemical shift trends in secondary structural motifs

S4. A visual example of the binomial test for *DR*_Cα-Hα_

S5. Worked-out example of a Student-based probability product (SBPP) calculation

S6. Benchmarking of SPIT via comparison to SSP and δ2D

S7. Extensions of the introduced techniques

S4.1. Application of SPIT to Cα-Cβ SCS differences

S4.2. Extension of the discordance principle to the Cα and Cβ atom types and application as a mis-referencing detection tool

**S1. Amino acid sequences of the model proteins used in discordance ratio (DR) and Structural Propensity Identification by *t*-statistics (SPIT) calculations**

yUBI: MQIFVKTLTGKTITLEVESSDTIDNVKSKIQDKEGIPPDQQRLIFAGRQLEDGRTLSDYNIQKESTLHLVLRLRGG

α-syn: MDVFMKGLSKAKEGVVAAAEKTKQGVAEAAGKTKEGVLYVGSKTKEGVVHGVATVAEKTKEQVTNVGGAVVTGVTAVAQKTVEGAGSIAAATGFVKKDQLGKNEEGAPQEGILEDMPVDPDNEAYEMPSEEGYQDYEPEA

WIPc: GSSHHHHHHVDSPRSGPRPPLPPDRPSAGAPPPPPPSTSIRNGFQDSPCEDEWESRFYFHPISDLPPPEPYVQTTKSYPSKLARNESRSSSNRRERGAPPLPPIPRLEHHHHHH

p53TAD^1-60^: GSMEEPQSDPSVEPPLSQETFSDLWKLLPENNVLSPLPSQAMDDLMLSPNNIEQWFTEDPGP

**S2. Existing RCCS predictors in the scientific literature**

In calculations of DR and SPIT, we used 8 RCCS predictors that we welected based on the criteria of possesssing at least nearest neighbor correction and being theoretically applicable to most aqueous protein studies. However, there are various other RCCS libraries available. We have already collected and discussed these in detail in another publication but decided to repeat the table summarizing these for informational purposes here (Tabel S1). For more details on the historical and conceptual evolution of RCCS predictors, we refer the reader to our earlier publication: Kovács D, Bodor A. 2023. The influence of random-coil chemical shifts on the assessment of structural propensities in folded proteins and IDPs. *RSC Adv* 13:10182 - 10203.

Table S1. Summary table of available RCCS prediction techniques, their conceptual origin and correction terms involved.

| **Potenci** | **Prosecco** | **Kjaergaard** | **ncIDP** | **Camcoil** | Wang L. | RefDB | **Wang** | PSSI | **Schwarzinger** | Lukin | **Wishart** | Braun | CSI | Bundi | Richarz | Howarth | McDonald | Method | |
| --- | --- | --- | --- | --- | --- | --- | --- | --- | --- | --- | --- | --- | --- | --- | --- | --- | --- | --- | --- |
| 2018 | 2017 | 2011 | 2010 | 2009 | 2006 | 2003 | 2002 | 2002 | 2000 | 1997 | 1995 | 1994 | 1992, 1994 | 1979 | 1978 | 1978 | 1969 | Year | |
| IDPs (ncIDP extended) | IDPs (selected BMRB) | Small peptides | IDPs (mostly BMRB) | Loop regions of globular proteins (selected BMRB) | Proteins from refDB | Selected BMRB data (database) | Database (selected BMRB) | Database (selected BMRB) | Small peptides, in acidic 8 M urea | Database (BMRB and literature) | Small peptide (GG-X-A/P-GG) | Small peptide (GG-X-A) | Globular proteins | Small peptide (GG-X-A) | Small peptide (GG-X-A) | Peptides and denatured proteins in D_2_O | Free amino acids, different small peptides | Type of system | |
| ✓ | ✓ | ✓ | ✓ | ✓ | 🗶 | 🗶 | ✓ | 🗶 | ✓ | 🗶 | ✓ | ✓ | 🗶 | 🗶 | 🗶 | 🗶 | 🗶 | Sequence | Corrections |
| ✓ | 🗶 | ✓ | 🗶 | 🗶 | 🗶 | 🗶 | 🗶 | 🗶 | 🗶 | 🗶 | 🗶 | 🗶 | 🗶 | 🗶 | 🗶 | 🗶 | 🗶 | Temperature |  |
| ✓ | ✓ | ✓ | 🗶 | ✓ | 🗶 | 🗶 | 🗶 | 🗶 | 🗶 | 🗶 | 🗶 | ✓ | 🗶 | 🗶 | 🗶 | 🗶 | 🗶 | pH |  |
| ✓ | ✓ | ✓ | ✓ | ✓ | 🗶 | ✓ | ✓ | ✓ | ✓ | ✓ | ✓ | 🗶 | ✓ | ✓ | 🗶 | 🗶 | 🗶 | H^N^ | Atom types |
| ✓ | ✓ | ✓ | ✓ | ✓ | 🗶 | ✓ | ✓ | ✓ | ✓ | ✓ | ✓ | 🗶 | ✓ | ✓ | 🗶 | 🗶 | ✓ | H^α^ |  |
| ✓ | ✓ | ✓ | ✓ | ✓ | ✓ | ✓ | ✓ | ✓ | ✓ | ✓ | ✓ | 🗶 | ✓ | 🗶 | ✓ | ✓ | 🗶 | C^α^ |  |
| ✓ | ✓ | ✓ | ✓ | ✓ | ✓ | ✓ | ✓ | ✓ | 🗶 | ✓ | ✓ | 🗶 | ✓ | 🗶 | ✓ | ✓ | 🗶 | C^β^ |  |
| ✓ | ✓ | ✓ | ✓ | ✓ | 🗶 | ✓ | ✓ | ✓ | ✓ | ✓ | ✓ | 🗶 | ✓ | 🗶 | ✓ | ✓ | 🗶 | C’ |  |
| ✓ | ✓ | ✓ | ✓ | ✓ | 🗶 | ✓ | ✓ | ✓ | ✓ | ✓ | ✓ | ✓ | ✓ | 🗶 | 🗶 | 🗶 | 🗶 | N |  |

**S3. Tabular summary of chemical shift trends in secondary structural motifs**

The tendencies for the chemical shifts of the six canonical atom-types utilized of protein NMR studies in different secondary structural motifs have long been well-known and were concisely summarized by Borcherds and Daughdrill (Borcherds and Daughdrill 2018). This information can be seen in Table S2.

Table S2. NMR chemical shift trend of the six canonical atom-types in helical and extended structural motifs

|  | Helical | Extended (β) |
| --- | --- | --- |
| Cα | + | – |
| C’ | + | – |
| Cß | – | + |
| Hα | – | + |
| HN | – | + |
| N | – | + |

**S4. A visual example of the binomial test for *DR_Cα_*_-Hα_**

The calculation of the discordance ratio and the corresponding binomial test are shown in Figure S1.


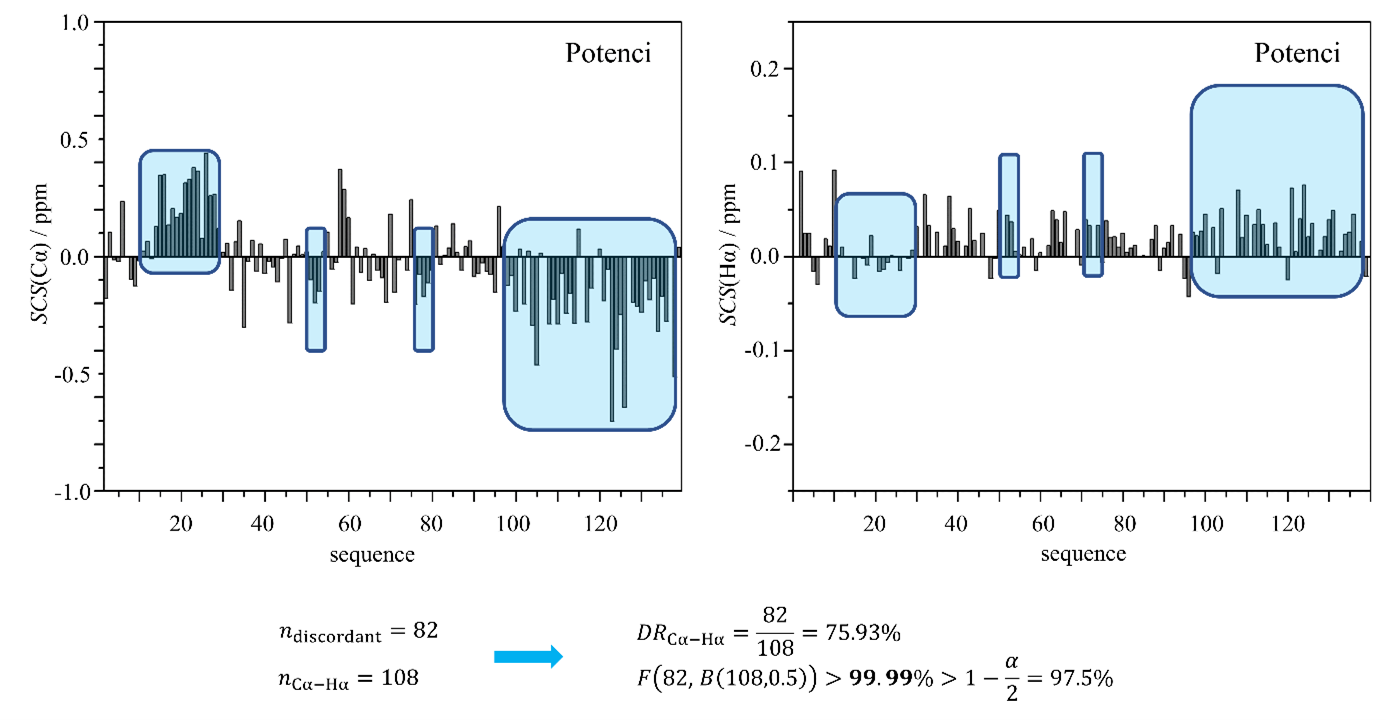


Figure S1. Example calculation scheme of the Cα-Hα discordance ratio and the corresponding binomial test for α-synuclein using the Potenci RCCS predictor. There are 82 discordant Cα-Hα SCS pairs out of the 108 instances with assignment for both atom types. This yields an empirical *DR*_Cα-Hα_ value of 75,93%, which is significantly larger than 50.00% at a 5% level of significance because the corresponding cumulative distribution function (denoted B(⸱)) value is 99.99% > 97.5%. Regions where the discordance principle is spectacularly fulfilled are highlighted.

**S5. Worked-out example of an SBPP calculation**

Below, we give an example of applying Structural Propensity Identification by t-statistics (SPIT) approach. We calculate the Student’s *t*-distribution-based probability products (SBPP) for the S9-A30 region of α-synuclein with experimental chemical shifts from BMRB 18857, at pH = 7.10 and *T* = 283 K.

After selecting the region of interest from the average SCS plot of the main text (Figure 5), the calculation starts by taking the corresponding sub-matrix of SCSs in Table S3.

Table S3. Sub-matrix of SCS values with the 7 selected predictors for the S9-A30 region of α-synuclein.

|  | Wishart | Wang | Camcoil | Kjaergaard | ncIDP | Potenci | Prosecco |
| --- | --- | --- | --- | --- | --- | --- | --- |
| S9 | 0.061 | 0.151 | 0.154 | -0.002 | 0.068 | -0.126 | -0.115 |
| K10 | 0.205 | -0.065 | 0.217 | 0.032 | 0.025 | -0.019 | -0.036 |
| A11 | 0.225 | 0.255 | 0.247 | 0.171 | 0.073 | 0.023 | -0.065 |
| K12 | 0.236 | -0.094 | 0.259 | -0.002 | -0.066 | 0.065 | 0.103 |
| E13 | 0.168 | 0.058 | 0.289 | -0.007 | -0.077 | -0.009 | 0.030 |
| G14 | 0.213 | -0.027 | -0.516 | 0.042 | 0.120 | 0.127 | 0.182 |
| V15 | 0.429 | 0.839 | 0.74 | 0.304 | 0.496 | 0.346 | 0.420 |
| V16 | 0.350 | 0.640 | 0.773 | 0.304 | 0.315 | 0.349 | 0.400 |
| A17 | 0.129 | 0.259 | -0.094 | 0.278 | 0.142 | 0.135 | 0.013 |
| A18 | 0.270 | 0.250 | 0.023 | 0.301 | 0.197 | 0.205 | 0.195 |
| A19 | 0.347 | 0.147 | 0.17 | 0.196 | 0.158 | 0.167 | 0.038 |
| E20 | 0.192 | 0.062 | 0.394 | 0.203 | 0.123 | 0.182 | 0.221 |
| K21 | 0.568 | 0.448 | 0.591 | 0.349 | 0.307 | 0.314 | 0.276 |
| T22 | 0.565 | 1.055 | 0.831 | 0.437 | 0.386 | 0.330 | 0.310 |
| K23 | 0.606 | 0.096 | 0.339 | 0.332 | 0.349 | 0.378 | 0.456 |
| Q24 | 0.608 | 0.368 | 0.291 | 0.251 | 0.273 | 0.364 | 0.293 |
| G25 | 0.261 | -0.099 | -0.474 | 0.172 | 0.214 | 0.077 | 0.127 |
| V26 | 0.484 | 0.874 | 0.900 | 0.298 | 0.524 | 0.441 | 0.482 |
| A27 | 0.358 | 0.308 | 0.204 | 0.293 | 0.255 | 0.259 | 0.132 |
| E28 | 0.298 | 0.178 | 0.609 | 0.334 | 0.274 | 0.265 | 0.305 |
| A29 | 0.231 | 0.191 | 0.016 | 0.239 | 0.148 | 0.118 | 0.030 |
| A30 | 0.321 | 0.251 | 0.156 | 0.025 | 0.061 | 0.017 | -0.058 |

At this point it is possible to calculate the (row) average (*m_i_*(*SCS*)) and the corresponding standard deviation (*s_i_*) values.

Table S4. Row averages and row standard deviations of Table S3.

|  | Average (*m_i_*(*SCS*)) | Standard deviation (*s_i_*) |
| --- | --- | --- |
| S9 | 0.061 | 0.114568 |
| K10 | 0.205 | 0.114188 |
| A11 | 0.225 | 0.124292 |
| K12 | 0.236 | 0.138413 |
| E13 | 0.168 | 0.12442 |
| G14 | 0.213 | 0.249977 |
| V15 | 0.429 | 0.202208 |
| V16 | 0.350 | 0.183766 |
| A17 | 0.129 | 0.130544 |
| A18 | 0.270 | 0.090138 |
| A19 | 0.347 | 0.091246 |
| E20 | 0.192 | 0.102771 |
| K21 | 0.568 | 0.129505 |
| T22 | 0.565 | 0.282634 |
| K23 | 0.606 | 0.15325 |
| Q24 | 0.608 | 0.122163 |
| G25 | 0.261 | 0.254622 |
| V26 | 0.484 | 0.227 |
| A27 | 0.358 | 0.073627 |
| E28 | 0.298 | 0.135188 |
| A29 | 0.231 | 0.090093 |
| A30 | 0.321 | 0.137244 |

With the data of Table S4, the *t*-values of Table S5 can be calculated.

Table S5. *t*-values corresponding to Table S3 and S4, calculated according to Equation 3 of the main text.

|  | *t*-values (*t_i_*) |
| --- | --- |
| S9 | 0.630115 |
| K10 | 1.188297 |
| A11 | 2.825027 |
| K12 | 1.368077 |
| E13 | 1.373093 |
| G14 | 0.213192 |
| V15 | 6.680483 |
| V16 | 6.439752 |
| A17 | 2.495746 |
| A18 | 6.042385 |
| A19 | 5.065960 |
| E20 | 5.064234 |
| K21 | 8.326553 |
| T22 | 5.234170 |
| K23 | 6.303941 |
| Q24 | 7.573928 |
| G25 | 0.412666 |
| V26 | 6.665174 |
| A27 | 9.286493 |
| E28 | 6.326975 |
| A29 | 4.082018 |
| A30 | 2.128809 |

According to the scheme of Figure 2 (main text), the *t*-values of Table S5 enable the calculation of the individual *P_i_*(+) and *P_i_*(-) probability values for the S9-A30 region based on Equation 4 of the main text. Table S6. contains these probabilities.

Table S6. Individual non-negativity (*P_i_*(+)) values for the S9-A30 region of α-synuclein. The V15-Q24 sub-region, focused on in later calculations is highlighted in green. The probabilities for negativity (*P_i_*(-) values) are also presented for information.

|  | *P_i_*(+) | *P_i_*(-) |
| --- | --- | --- |
| S9 | 0.724077 | 0.275923 |
| K10 | 0.860193 | 0.139807 |
| A11 | 0.984923 | 0.015077 |
| K12 | 0.889846 | 0.110154 |
| E13 | 0.890586 | 0.109414 |
| G14 | 0.580882 | 0.419118 |
| V15 | 0.999727 | 2.72517×10^-4^ |
| V16 | 0.999668 | 3.31702×10^-4^ |
| A17 | 0.976602 | 0.0233984 |
| A18 | 0.999535 | 4.64765×10^-4^ |
| A19 | 0.998851 | 0.00114856 |
| E20 | 0.998849 | 0.00115052 |
| K21 | 0.999919 | 8.14259×10^-5^ |
| T22 | 0.999025 | 9.74661×10^-4^ |
| K23 | 0.999628 | 3.71544×10^-4^ |
| Q24 | 0.999862 | 1.37657×10^-4^ |
| G25 | 0.652904 | 0.347096 |
| V26 | 0.999724 | 2.75898×10^-4^ |
| A27 | 0.999956 | 4.40981×10^-5^ |
| E28 | 0.999636 | 3.64416×10^-4^ |
| A29 | 0.996756 | 0.00324366 |
| A30 | 0.961332 | 0.0386682 |

Finally, the SBPP(+) value for the S9-A30 region (Table S7) is calculated as the product of all the non-negativity probability values in Table S6. As this is only 17.16%, not enough to assume the presence of a convincing transient helix over the entire region. However, looking at the individual *P_i_*(+), one is tempted to calculate the SBPP(+) for the V15-Q24 region, where all values are above 95%. This is done by multiplying the values with green background in Table S4 and the resulting 97.18% SBPP(+) is shown in Table S8. This is a convincing value, making the presence of a residual helix very likely. Calculating SBPP(-) values, although possible, is not relevant in a region with positive average SCS values.

Table S7. The final, calculated SBPP value for the S9-A30 region of α-synuclein.

|  | SBPP (+) |
| --- | --- |
| S9-A30 | 0.171573 |

Table S8. The final, calculated SBPP value for the V15-Q24 region of α-synuclein.

|  | SBPP (+) |
| --- | --- |
| V15-Q24 | 0.971794 |

**S6. Benchmarking of SPIT via comparison to SSP and δ2D**

Below, SPIT is compared to similar, albeit conceptually different computational methods: the secondary structural propensity score (SSP) (Marsh and others 2006) and δ2D (Camilloni and others 2012). The SSP computations for the Cα, Cβ and Hα atom-types were performed using the Perl script-based SSP version 1.0 (2006) software. The δ2D calculations were done as implemented online by the Vendruscolo lab at <https://www-cohsoftware.ch.cam.ac.uk//>.

In the case of yUBI, both the SSP plot in Figure S2A and the δ2D plot in S2B display large and sudden changes because of the sudden appearance of folded secondary structural motives along the amino acid sequence, as expected for a folded protein. However, the use of continuous lines covers up missing residues which biases the analysis, in contrast to SPIT. Using continuous lines also hinders identifying the start and termination of secondary structural motives, forcing one to evaluate individual probability values. Despite these limitations, both Figure S2A and S4B show the consensus structural motifs of yUBI with minor differences.

In contrast, the SSP score of the 3 IDPs never leaves the ± 0.5 interval (Figure S2C, E and D) and coil secondary structure dominates throughout their δ2D graphs (Figure S2D, F and H) with few exceptions. For α-syn both SSP and δ2D indicate almost complete disorder (Figure S2C and D). SSP displays mild signs of extended propensities in the C-terminal region which is hardly visible in the δ2D plot but was well identifiable based on SPIT (Figure 5) and has been described in the literature as discussed earlier. Neither SSP nor δ2D indicate the helical propensity in the S9-A30 segment, in contrast with SPIT. This could be because both SSP and δ2D assess structural strength between the complete disorder and fully folded structure, making them less sensitive to very weak but noticeable propensities. In contrast, SPIT does not consider any information about SCSs in properly folded structures but it filters out small yet significant SCS deviations from the ‘noise’ of complete disorder. Thus, SPIT is a more sensitive tool for IDPs – at the expense of assessing structural strength – than its alternatives.

WIPc is the only presented IDP for which SSP values outside ± 0.5 interval appear and the dominance of coil contributions is not complete in the δ2D plot. SSP indicates a β-propensity in the H9-P20 segment, with 4 prolines out of 12 residues. This also appears in δ2D but increase in β-contribution is weaker. Proline-rich regions are especially challenging for both techniques for different reasons. In SSP the default RCCS library is that of RefDB, lacking neighbor corrections. Therefore, residues preceding prolines are omitted. This introduces gaps in SSP plots in proline-rich regions, which is only visually attenuated by continuous line plotting. Conversely, δ2D uses the Camcoil RCCS predictor, challenging its nearest neighbor corrections in poly-proline segments.

In the P31-P36 segment of WIPc – containing 6 consecutive proline residues – β-propensity is indicated by δ2D (Figure S2F). However, this β-propensity is probably an artefact caused by the insufficient proline correction of Camcoil RCCSs. This region is completely missing in Figure S4E because of default residue omission in SSP. The same holds for the A98-P103 segment of WIPc with 4 prolines out of 6 residues. In SPIT false identification of such propensities is attenuated by using multiple well-selected RCCS predictors, *e.g.* having reliable nearest neighbor correction terms for proline.

Regarding the central helical region of WIPc at around P48-Y58, SSP displays a mild helical propensity, more convincingly identified by SPIT above. The δ2D plot shows a very weak increase of helical propensity in this region, which would probably be deemed irrelevant by looking at δ2D alone.

In the case of p53TAD^1-60^, the helix between residues T18 and L25 appears (although still with SSP scores below 0.5) but the beginning and termination are difficult to judge (Figure S2G). Also, the window-averaging of SCSs that is applied in SSP smooths out single, large SCSs, which might be important in the case of IDPs. This is a disadvantage compared to SPIT, where such individual values are not smoothed out by the potentially smaller amplitude SCSs of neighboring amino acids.

The SSP plot of p53TAD^1-60^ (Figure S2G) might also be interpreted to indicate 2 very mildly helical stretches between residues 30 and 50, possibly separated by a mildly extended region. This complies with SPIT results. The first transient helix is also well indicated by δ2D but the second pair of such motifs is not. On the contrary, an increase of β-contribution is observed between residues L45 and D48 in Figure S2H.


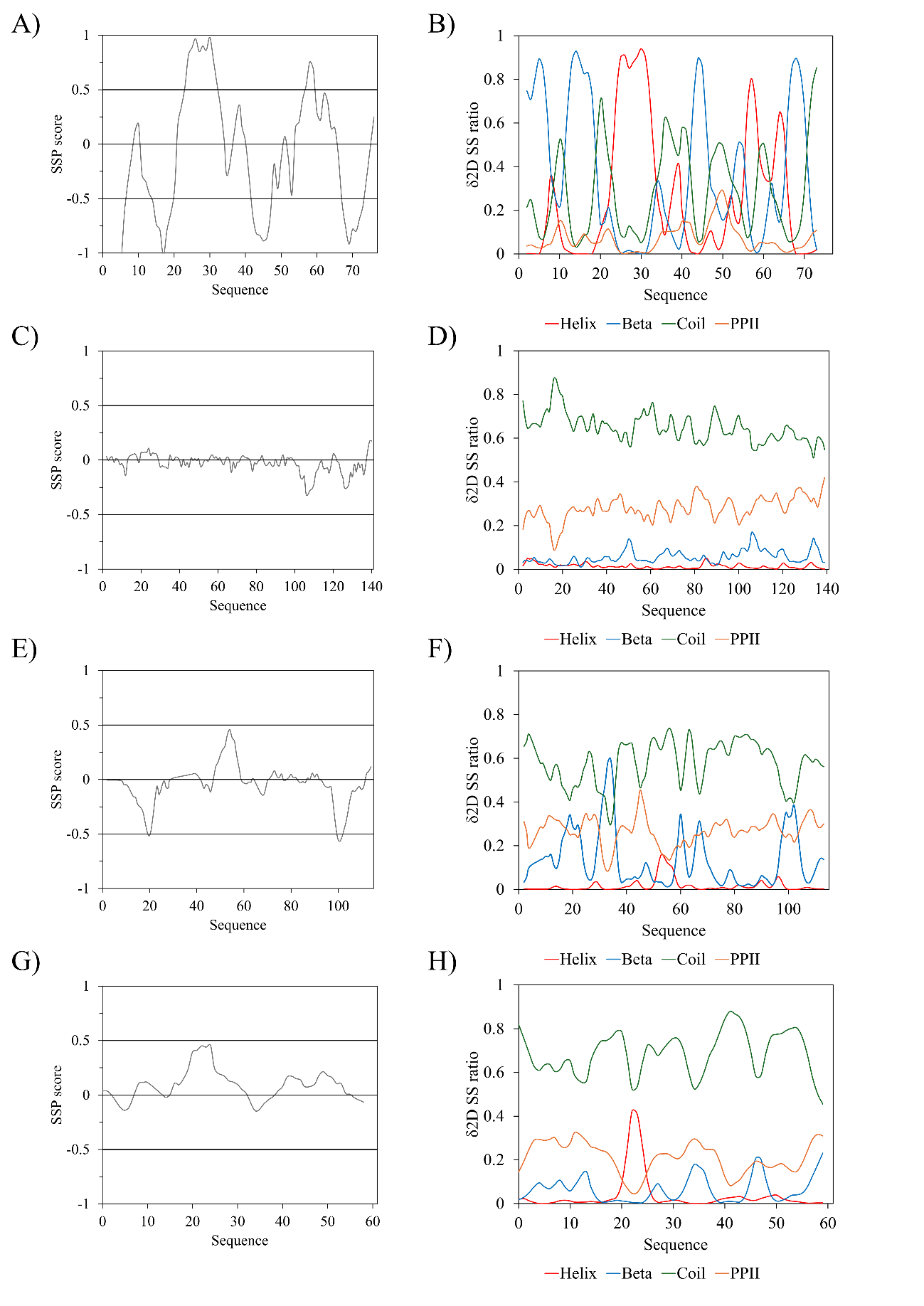


Figure S2. Respective SSP and δ2D plots of the four model proteins. A-B): yUBI, C-D): α-syn, E-F): WIPc and G-H): p53TAD^1-60^. The ±0.5 SSP limits – outside which a strong structural propensity is generally accepted are indicated by horizontal lines. In the δ2D plots the structural contribution of the four secondary motives included in the method are shown in red (helix), blue (beta), green (coil) and polyproline II (PPII, orange).

Altogether, despite their fundamental differences, the three approaches agree on most structural features in the 4 model proteins with SPIT being more sensitive and locally exact in the case of IDPs, especially proline-rich WIPc and p53TAD^1-60^.

**S7. Extensions of the introduced techniques**

Both introduced techniques, *i.e.* SPIT and Cα-Hα discordance calculations can be extended by including other atom types than in the main text. Such applications are shown in the following sub-chapters.

**S7.1 Application of SPIT to Cα-Cβ SCS differences**

Instead of using SCSs of a single atom type, sometimes the difference of the Cα and Cβ SCSs is plotted against the sequence. The introduced SPIT approach can be directly applied in such cases without any modification. Below, we demonstrate this using the chemical shift data of yUBI as an example in Figure S3.


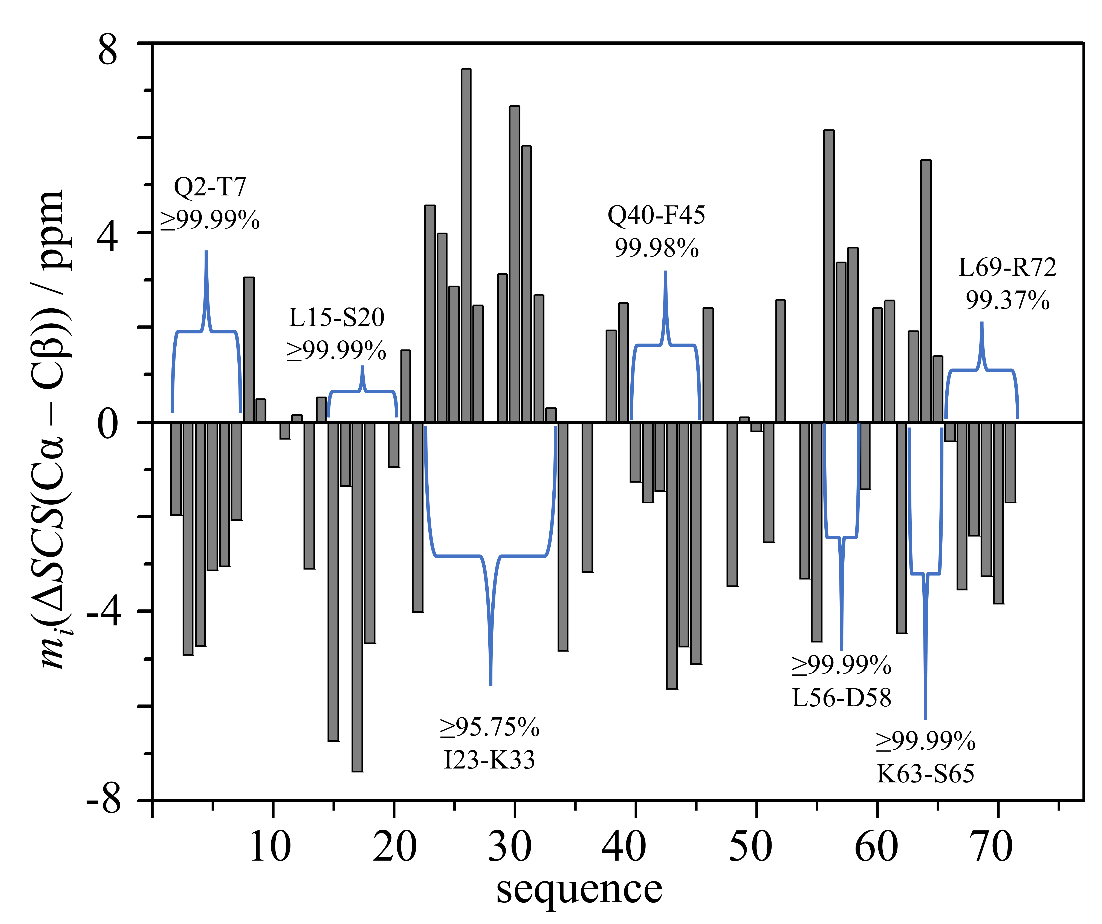


Figure S3. Average Cα-Cβ difference SCS plot of yUBI. Regions of interest are highlighted by curly brackets.

The SBPP values calculated for Cα-Cβ difference SCSs can be interpreted the same way as in the case of Cα. The first 2 β-sheets and the long helix of yUBI appear exactly in the same regions as indicated by Cα SPIT analysis: Q2-T7, L15-S20 and I23-K33, respectively. Note, that the missing assignment of the Cβ chemical shift for S28 causes a ‘gap’ in the positive pattern (Figure S3). The main difference between simple Cα and the Cα-Cβ difference results is the next extended structure, indicated between Q40 and F45 by Cα-Cβ difference SPIT analysis. This is more in line with the 2 PDB structures (1D3Z and 1UBQ) mentioned in the main text, than the R42-A46 segment indicated by SPIT for Cα. However, no double extended motif is observed for R48-E51 based on Cα-Cβ differences. Another difference is that SPIT analysis of for Cα-Cβ differences seems to indicate a short K63-S65 helix, however this is probably an artifact resulting from the presence of the following and well-identified L69-R72 sheet motif. The short L56-D58 helix also matches the Cα results perfectly.

Generally larger SCS values and an increased sensitivity towards β-motifs are advantages of Cα-Cβ differences over their single atom type counterparts. However, the limited availability of Cβ chemical shift data and the lack of this atom type in glycines are disadvantages.

**S7.2. Extension of the discordance principle to the Cα and Cβ atom types and application as a mis-referencing detection tool**

Similarly to the Hα environment, the chemical shift of Cβ changes in the opposite direction as that of Cα. Therefore, Cα-Cβ discordance ratios can be used in the same way as Cα-Hα discordance ratios shown in the main text. In addition, DR values can have broader applications than just a self-consistency-based prefiltering for RCCS predictors. Another advantage of DR values is that they generally highlight inconsistencies in the SCS dataset and can be used to detect mis-referencing.

In Figure S4, we show the effect of intentionally mis-referencing all ^13^C chemical shifts of yUBI by 0.5 and 2.0 ppm.


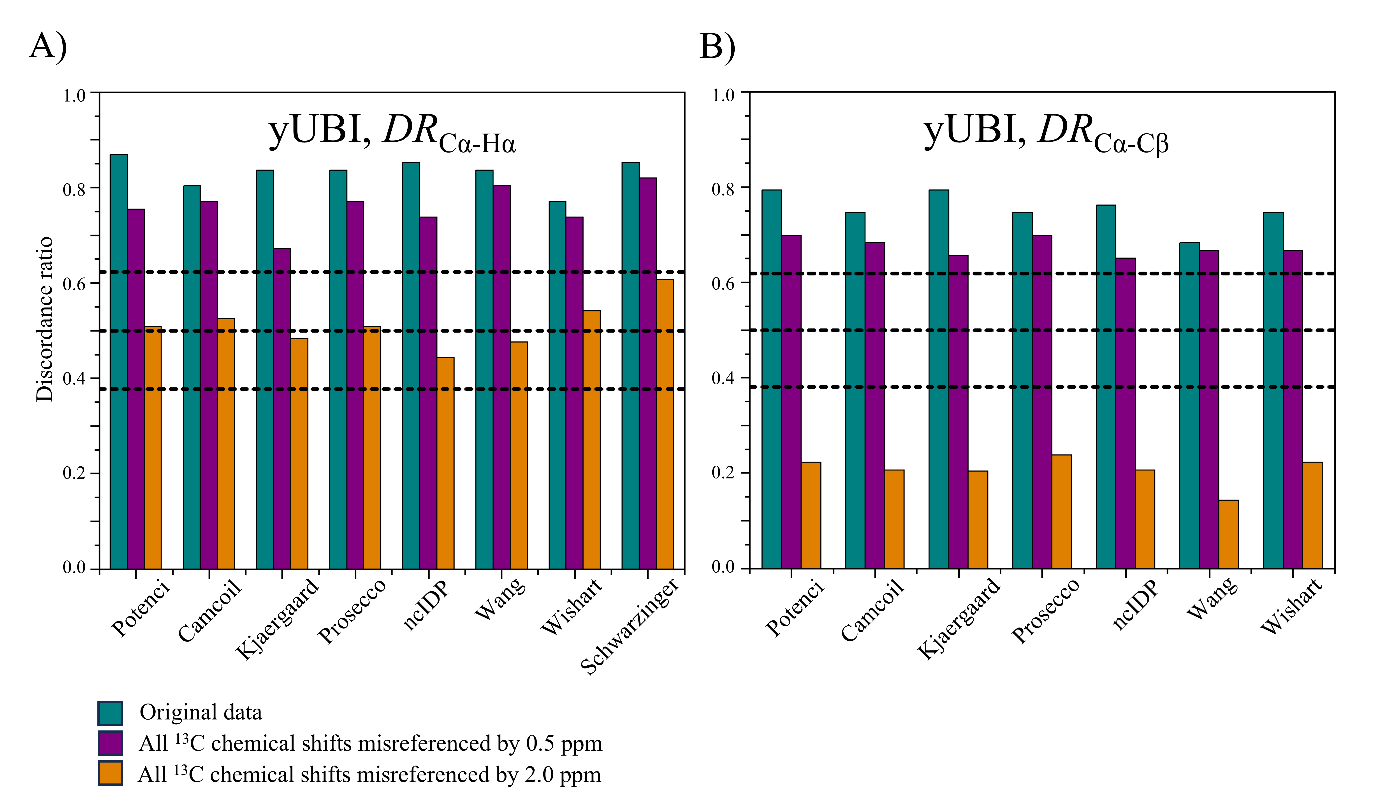


Figure S4. (A) *DR*_Cα-Hα_ (B) *DR*_Cα-Cβ_ values for yUBI with all ^13^C chemical shifts intentionally mis-referenced by 0 (original dataset, green), 0.5 (purple) and 2.0 (orange) ppm, respectively. The Schwarzinger RCCS dataset is excluded in (B) because it lacks the Cβ atom type. The black horizontal lines from top to bottom correspond to the 97.5%, 50% and 2.5% probability limits of the appropriate binomial distribution as in the main text.

Figure S4 shows that both *DR*_Cα-Hα_ and *DR*_Cα-Cβ_ values indicate mis-referencing. In the former case, all discordance ratios fall into the insignificant region when 2.0 ppm mis-referencing is applied (Figure S4A). In the case of *DR*_Cα-Cβ_ values, the effect is even more pronounced: all discordance ratios become significantly worse than 0.5 in the case of 2.0 ppm mis-referencing (Figure S4B). This is understandable because in the second case both atom types (Cα and Cβ) are affected by mis-referencing while Hα is not. This demonstrates that *DR*_Cα-Cβ_ analysis is a simple yet exceptionally powerful tool for the detection of mis-referencing.
